# Attitudes and Factors Associated With Intention to the Third Dose of COVID-19 Vaccine Among Adolescents: A Cross-Sectional Survey in 3 Provinces of China

**DOI:** 10.1017/dmp.2022.181

**Published:** 2022-07-27

**Authors:** Taishun Li, Ruowen Qi, Yi-Hua Zhou, Yuqian Luo, Shi-Yuan Wang, Bingwei Chen, Biyun Xu

**Affiliations:** 1 Medical Statistics and Analysis Center, Nanjing Drum Tower Hospital, Nanjing University Medical School, Nanjing, China; 2 Department of Laboratory Medicine and Infectious Diseases, Nanjing Drum Tower Hospital, Nanjing University Medical School, Nanjing, China; 3 School of Public Health, Southeast University, Nanjing, China

**Keywords:** COVID-19, vaccine, third dose, adolescents, China

## Abstract

**Objective:**

The COVID-19 caused a world pandemic, posing a huge threat to global health. Widespread vaccination is the most effective way to control the pandemic. Vaccination with the third dose of the COVID-19 vaccine is currently underway. We aimed to determine the attitude of adolescents toward the third dose of COVID-19 vaccine.

**Methods:**

A structured questionnaire was administered between 16 August and 28 October 2021 among adolescents aged 12–17 years in three provinces of eastern region of China based on convenience sampling. The questionnaire was specifically developed to assess the adolescents’ attitude toward and willingness to accept a third dose of the COVID-19 vaccine.

**Results:**

In total, 94.3% (1742/1847) of the adolescents intended to accept the third dose of the COVID-19 vaccine. Age between 15–17 years, no worry about vaccine safety, confidence for vaccine effectiveness, and supporting opinion from parents were independently associated with acceptance of the third dose (*p* < 0.05).

**Conclusions:**

It is necessary for governments and school administrators to raise adolescents’ and parents’ awareness of the benefits and safety of the third dose of vaccination, which should be effective to increase the vaccination coverage among adolescents.

The coronavirus disease 2019 (COVID-19) has caused a world pandemic, posing a huge threat to the health of the people, the national economy, and the stability of the world.^1,2^ By establishing higher levels of herd immunity, widespread vaccination is the most effective way to control the pandemic of COVID-19. The vaccination of COVID-19 vaccine for adolescents aged 12-17 y in China has been started since July 2021. However, the effectiveness of the vaccine against COVID-19 gradually decreases, with the waning of immunity over time.^3,4^ The third vaccine dose, referred to as a booster, is proved to be effective to overcome this issue, and studies have shown that the third dose of COVID-19 vaccine is safe and effective.^5^

Numerous factors affect the acceptance of the third dose of COVID-19 vaccine during the pandemic, especially among adolescents. This study aimed to determine the attitude and intention factors for the third dose of COVID-19 vaccine. Understanding the willingness and attitudes of adolescents toward the third dose could help policy-makers identify appropriate interventions to alleviate worries and inform public health decisions about the COVID-19 vaccination.

## Methods

### Study Design

A cross-sectional questionnaire was developed and distributed among adolescents aged 12-17 y in 3 provinces (Anhui, Fujian, and Jiangsu) of the eastern region of China from August 16 to October 28, 2021, and 1 senior high school and 1 junior high school in each province were selected. The protocol was approved by the Ethics Committee of the Nanjing Drum Tower Hospital (#2021-462-01). The informed consent was indicated at the beginning of the questionnaire, which was consented by the parents or guardians of the participants.

### Measures

The survey consisted of questions as follows: (1) demographic information; (2) knowledge of and attitude to COVID-19 vaccine; (3) willingness to accept the third dose. The detailed questions in English of our questionnaire are provided in Supplementary File 1 (Text S1).

### Statistical Analysis

Descriptive analysis of the demographics was expressed by means and standard deviations or proportions. To compare the main variables between groups, independent t-tests were performed to analyze the continuous variables, and chi-squared tests were performed to analyze the categorical variables. Multivariable logistic regression analysis was performed, including all factors showing significance (*P* < 0.05) in the univariate analysis, to explore the factors associated with the willingness to accept the third dose of COVID-19 vaccine. All statistical analyses were performed using the R software (R version 4.04). A *P* value of ≤ 0.05 was considered statistically significant.

## Results

### Demographic Characteristics

The survey was released to 2100 adolescents, and a total of 2048 (97.5%) responded. Of them, 202 (9.8%) who did not complete survey questionnaire were excluded. Finally, 1847 participants who completed survey questionnaire were included in this study. There were 48.2% (834/1846) males and 60.9% (1110/1822) from rural areas. Overall, 94.3% (1742/1847) adolescents intended to accept the third vaccine dose, and 5.7% (105/1847) did not. The results indicated that the acceptance willingness was associated with age (15-17 y), region (Anhui province), district (rural), and educational level (senior high school). Participants who had been quarantined or affected by COVID-19 had more intention to get vaccinated (*P* < 0.05). In addition to these differences, the confidence for the vaccine safety and effectiveness, and parents’ opinions did affect adolescents’ view on vaccination (*P* < 0.05). The detailed comparisons of the 2 groups are summarized in Table 1.

**Table 1.** Demographic characteristics of the participants in the survey

		Intent to accept the 3^rd^ vaccine dose, n (%)		
Variables	Total N (%)	Yes	No	*p*
Overall	1847 (100.0)	1742 (94.3)	105 (5.7)	
Gender	1846 (99.9)			0.280
Female		907 (52.1)	49 (46.7)	
Male		834 (47.9)	56 (53.3)	
Age	1847 (100.0)			<0.001*
12-14 years		770 (44.2)	69 (65.7)	
15-17 years		972 (55.8)	36 (34.3)	
Province	1847 (100.0)			<0.001*
Anhui		565 (32.4)	14 (13.3)	
Jiangsu		852 (48.9)	60 (57.2)	
Fujian		325 (18.7)	31 (29.5)	
Location	1822 (98.6)			0.008*
Rural		1060 (61.7)	50 (48.5)	
Urban		659 (38.3)	53 (51.5)	
Education Level	1847 (100.0)			<0.001*
Junior high school		920 (52.8)	81 (77.1)	
Senior High school		822 (47.2)	24 (22.9)	
Had been quarantined because of the COVID-19	1846 (99.9)			0.036*
Yes		38 (2.2)	6 (5.7)	
No		1703 (97.8)	99 (94.3)	
Is life affected by the COVID-19	1843 (99.8)			0.001*
Seriously affected		230 (13.2)	19 (18.1)	
Affected		896 (51.6)	35 (33.3)	
No effect		612 (35.2)	51 (48.6)	
If medical workers in the family	1803 (97.6)			0.987
Direct relatives		138 (8.1)	8 (7.8)	
Other relatives		355 (20.9)	22 (21.4)	
No		1207 (70.0)	73 (70.9)	
Worry about vaccine safety	1844 (99.8)			<0.001*
Worry		233 (13.4)	39 (37.1)	
Not worry		1506 (86.6)	66 (62.9)	
Confidence for vaccine effectiveness	1845 (99.9)			<0.001*
Confident		1674 (96.2)	79 (75.2)	
No confidence		66 (3.8)	26 (24.8)	
Parents accepted the COVID-19 vaccine	1844 (99.8)			0.002*
Both		1619 (93.1)	88 (83.8)	
Neither		12 (0.7)	2 (1.9)	
Either of two		108 (6.2)	15 (14.3)	
Parents’ opinion about adolescents’ vaccination	1846 (99.9)			<0.001*
Support		1665 (95.6)	76 (72.4)	
Neutral		52 (3.0)	14 (13.3)	
Oppose		2 (0.1)	6 (5.7)	
Unknown		22 (1.3)	9 (8.6)	

*P < 0.05.

### Factors Associated With the Third Dose of the COVID-19 Vaccine Acceptance

Multivariate logistic regression for willingness to take the third dose of the COVID-19 vaccine is shown in Table 2. Age between 15 and 17 y, no worry about vaccine safety, confidence for vaccine effectiveness, and supporting opinion from parents were independently associated with acceptance of the third dose (*P* < 0.05).

**Table 2.** Factors of participants’ willingness to accept the third dose of the COVID-19 vaccine by multivariate logistic regression analysis

Variables	*β*	SE	OR (95% CI)	*p*
Age group (year)				
12-14			Ref	
15-17	0.81	0.23	2.26 (1.43-3.55)	0.001*
Worry about vaccine safety				
Worry			Ref	
Not worry	1.04	0.24	2.82 (1.75-4.54)	<0.001*
Confidence for vaccine effectiveness				
Confident			Ref	
No confidence	−1.43	0.32	0.24 (0.13-0.45)	<0.001*
Parents’ opinion of children’s vaccination				
Support			Ref	
Neutral	−1.35	0.38	0.26 (0.12-0.55)	0.001*
Oppose	−2.10	1.00	0.12 (0.02-0.87)	0.036*
Unknown	−1.15	0.50	0.32 (0.12-0.84)	0.021*

*P < 0.05.

## Discussion

This study was conducted to investigate the willingness of adolescents at the age of 12-17 y to take the third dose of the COVID-19 vaccine. This study indicated that 94.3% (1742/1847) of participants would like to accept the third dose in the future. The main influencing factors of adolescents’ willingness to accept the third dose were age, concerns about vaccine safety and effectiveness, and parents’ opinions.

Hesitancy to receive vaccination against COVID-19 has been widely reported.^6^ The main reasons for the third vaccine dose hesitancy in adolescents found in the present study were concerns about the safety and effectiveness of vaccines, which is consistent with that in other reports.^7,8^ Hence, the government should focus on the public awareness of the safety and effectiveness of COVID-19 vaccines.

The results found significant associations between parents’ attitudes and COVID-19 vaccine acceptance willingness of the third dose in adolescents, which is in accordance with the previous study that focused on parents’ willingness to accept the COVID-19 vaccine for their children.^9^ This may be due to the fact that adolescents are minors and their behavior is influenced by parents. The results also indicated that vaccinated parents were more supportive of their children getting the third vaccine dose than unvaccinated parents. Recent research reported that adolescents have the capacity to understand benefit and safety concerns about the COVID-19 vaccination, and suggested COVID-19 vaccination of minors without parental consent.^10^ In the future, public health agencies should stress the safety and effectiveness of vaccines to dismiss the parents’ concerns and improve parents’ own vaccine acceptance rate, which will promote adolescents to get the third dose.

## Supplementary material

For supplementary material accompanying this paper visit https://doi.org/10.1017/dmp.2022.181. click here to view supplementary material
