# Supplementary material for: Attitudes and Factors Associated With Intention to the Third Dose of COVID-19 Vaccine Among Adolescents: A Cross-Sectional Survey in 3 Provinces of China
Source: Disaster Med Public Health Prep. 2022 Jul 27:1–4. doi: 10.1017/dmp.2022.181 (PMC9530370; doi:10.1017/dmp.2022.181)
Supplement: Supplementary file 1 [file S1935789322001811sup001.docx]

**Supplementary Materials:**

Supplementary File 1: Text S1. The detailed English version of questionnaire
